# A New Drug Design Targeting the Adenosinergic System for Huntington's Disease

**DOI:** 10.1371/journal.pone.0020934

**Published:** 2011-06-21

**Authors:** Nai-Kuei Huang, Jung-Hsin Lin, Jiun-Tsai Lin, Chia-I Lin, Eric Minwei Liu, Chun-Jung Lin, Wan-Ping Chen, Yuh-Chiang Shen, Hui-Mei Chen, Jhih-Bin Chen, Hsing-Lin Lai, Chieh-Wen Yang, Ming-Chang Chiang, Yu-Shuo Wu, Chen Chang, Jiang-Fan Chen, Jim-Min Fang, Yun-Lian Lin, Yijuang Chern

**Affiliations:** 1 National Research Institute of Chinese Medicine, Taipei, Taiwan; 2 Division of Mechanics, Research Center for Applied Sciences, Academia Sinica, Taipei, Taiwan; 3 Institute of Biomedical Sciences, Academia Sinica, Taipei, Taiwan; 4 School of Pharmacy, National Taiwan University, Taipei, Taiwan; 5 Department of Chemistry, National Taiwan University, Taipei, Taiwan; 6 The Genomics Research Center, Academia Sinica, Taipei, Taiwan; 7 Graduate Institute of Biotechnology, Chinese Culture University, Taipei, Taiwan; 8 Department of Neurology, Boston University School of Medicine, Boston, Massachusetts, United States of America; Institut National de la Santé et de la Recherche Médicale, France

## Abstract

**Background:**

Huntington's disease (HD) is a neurodegenerative disease caused by a CAG trinucleotide expansion in the Huntingtin (Htt) gene. The expanded CAG repeats are translated into polyglutamine (polyQ), causing aberrant functions as well as aggregate formation of mutant Htt. Effective treatments for HD are yet to be developed.

**Methodology/Principal Findings:**

Here, we report a novel dual-function compound, *N* ^6^-(4-hydroxybenzyl)adenine riboside (designated T1-11) which activates the A_2A_R and a major adenosine transporter (ENT1). T1-11 was originally isolated from a Chinese medicinal herb. Molecular modeling analyses showed that T1-11 binds to the adenosine pockets of the A_2A_R and ENT1. Introduction of T1-11 into the striatum significantly enhanced the level of striatal adenosine as determined by a microdialysis technique, demonstrating that T1-11 inhibited adenosine uptake *in vivo*. A single intraperitoneal injection of T1-11 in wildtype mice, but not in A_2A_R knockout mice, increased cAMP level in the brain. Thus, T1-11 enters the brain and elevates cAMP via activation of the A_2A_R *in vivo*. Most importantly, addition of T1-11 (0.05 mg/ml) to the drinking water of a transgenic mouse model of HD (R6/2) ameliorated the progressive deterioration in motor coordination, reduced the formation of striatal Htt aggregates, elevated proteasome activity, and increased the level of an important neurotrophic factor (brain derived neurotrophic factor) in the brain. These results demonstrate the therapeutic potential of T1-11 for treating HD.

**Conclusions/Significance:**

The dual functions of T1-11 enable T1-11 to effectively activate the adenosinergic system and subsequently delay the progression of HD. This is a novel therapeutic strategy for HD. Similar dual-function drugs aimed at a particular neurotransmitter system as proposed herein may be applicable to other neurotransmitter systems (e.g., the dopamine receptor/dopamine transporter and the serotonin receptor/serotonin transporter) and may facilitate the development of new drugs for other neurodegenerative diseases.

## Introduction

Huntington's disease (HD) is an autosomal dominant neurodegenerative disease characterized by chorea, dementia, and psychiatric symptoms. As the disease progresses, concentration and short-term memory diminish and involuntary movements of the head, trunk, and limbs increase. Walking, speaking, and swallowing abilities deteriorate. Eventually, death results from complications such as choking, infection, or heart failure. The causative mutation is a CAG trinucleotide expansion in exon 1 of the Huntingtin gene (Htt) [1]. The normal Htt gene has 35 or fewer CAG repeats in its N-terminal region, whereas that of HD patients is associated with 36 or more repeats. The expanded CAG repeats are translated into polyglutamine residues (polyQ) in the Htt protein. When the number of CAG repeats exceeds 35, degeneration of several brain areas (particularly the striatum) occurs. Formation of Htt aggregates and alteration of overall gene expression profiles have also been reported in peripheral tissues, including blood cells, the liver, and the kidney [2], [3]. Drugs currently available for treating HD patients are mostly for symptom relief, and some have unfavorable side effects [4]. Effective treatments for HD are yet to be developed.

Adenosine is an important neuromodulator that links neuronal activity with energy metabolism [5]. Conditions that drain energy reserves or cause an energy imbalance, such as intensive exercise and ischemia, elevate adenosine levels [6]. There are four adenosine receptors (A_1_, A_2A_, A_2B_, and A_3_) and several adenosine transporters. Because of their expression profiles and affinities toward adenosine, the A_1_R and A_2A_R are believed to regulate important physiological functions in the brain. In particular, the A_2A_R has attracted attention as a potential drug target in HD because it is highly prevalent in the striatum, where mutant Htt causes early damage. In addition, evidence from various laboratories has clearly shown that tonic activation of the A_2A_R is required for the function of several important neurotrophic factors (including brain-derived neurotrophic factor, fibroblast growth factor, and glial cell line-derived neurotrophic factor) [7]–[9]. A_2A_-related drugs therefore have been implicated in the treatment of HD [10]–[16]. We previously reported that an A_2A_ agonist (CGS21680, CGS) significantly ameliorates several symptoms of HD (viz, brain atrophy, striatal aggregates, deteriorated motor coordination, and urea cycle deficiency) in a transgenic mouse model of HD [12], [13]. Nevertheless, certain adverse effects of currently available A_2A_ drugs (e.g., CGS) that exhibit high A_2A_R affinity prevent their clinical application [17]. In the present study, we describe a novel agonist [*N* ^6^-(4-hydroxybenzyl)adenine riboside (designated T1-11)] of the A_2A_R that also inhibits the adenosine transporter, and which may be used to treat HD without evident side effects because of its moderate affinity to its target molecules.

T1-11 was originally purified from *Gastrodia elata* (GE), a Chinese medicinal herb that has been used extensively in Asia for at least 1500 years. It is traditionally used to treat headaches, dizziness, limb numbness, and spasms, especially those of convulsive illnesses such as epilepsy and tetanus. Because of its efficacy in treating epileptic diseases, many studies have been performed to investigate its role in preventing neuronal damage. For example, gastrodin, a component of GE, was shown to alter GABA metabolism in the gerbil hippocampus [18]. The ether fraction of GE also significantly reduced neuronal cell death induced by β-amyloid [19]. We previously reported that two active components [T1-11 and bis(4-hydroxybenzyl)sulfide] purified from an aqueous methanolic extract of GE prevented apoptosis of serum-deprived PC12 cells by suppressing JNK activity [20], [21]. Herein, we demonstrate that T1-11 protects PC12 cells *in vitro* and also exerts a beneficial effect on symptom progression in a mouse model of HD via targeting two components of the adenosinergic mechanism.

## Results

### Purification of T1-11 from a Chinese herb

**Figure 1:**
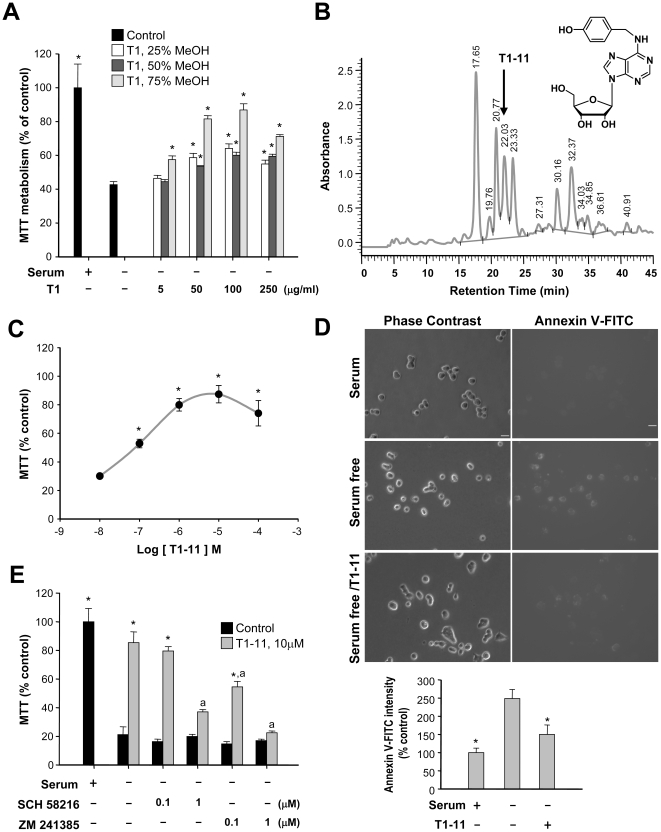
A fraction of the GE extract prevents serum-deprived PC12 cell apoptosis. (A, C, E) Serum-deprived PC12 cells were treated with or without the indicated reagent(s) for 24 h. Cell viability was expressed as a percentage of the MTT activity measured in the serum-containing group. Data points represent the mean ± s.e.m. of at least three independent experiments. *p<0.05, versus the corresponding serum-deprived group. ^a^ *p*<0.05, versus the corresponding serum-deprived/T1-11 treated group. (B) Chromatogram of active fractions of GE conducted by HPLC on a Merck RP-18e (250×4.6 mm) column. The position of T1-11 is indicated by an arrow. The structure of T1-11 is shown in the upper right corner. (D) Serum-deprived PC12 cells were treated with serum or T1-11 (10 µM) as indicated for 24 h, stained with annexin V-FITC, and analyzed using microscope and flow cytometry. The median values of FITC fluorescence intensities were collected using an FL-1 channel (bottom panel). Representative pictures of cells in each condition are shown. Bars:10 µm. Data points represent the mean ± s.e.m. of at least three independent experiments.

We previously reported that a fraction of GE prevents apoptosis in PC12 cells by activating the A_2A_R [20]. In the present study, we further purified the active component of this GE extract. The aqueous ethanolic extract of GE (∼15% yield based on dried weight) was subjected to Diaion HP-20 column chromatography using elution from H_2_O to MeOH gradients. As shown in Figure 1A, several fractions of the aqueous methanolic extract conferred protection against PC12 cell death induced by serum withdrawal. The most effective dosage was the 75% MeOH fraction, which was subjected to further fractionation and purification using Sephadex LH-20 column chromatography (Fig. 1B). Sixteen known compounds (including gastrodin, 4-hydroxybenzaldehyde, and parishin) and one previously uncharacterized component (T1-11) were identified [22]–[24]. Of these compounds, T1-11 was considered the most promising because it is an adenosine analogue (Fig. 1B). HPLC was used to monitor the chemical profiles of various batches based on their ability to prevent cell death induced by serum withdrawal. Chromatograms of the active fractions showed that the retention time for T1-11 was 22.03 min (Fig. 1B; Supplementary Figs. S1, S2). T1-11 is a colorless amorphous powder with a molecular formula of C_17_H_20_O_5_N_5_. Spectral methods (HR-FAB-MS, IR, ^1^H, and ^13^C NMR) determined that the structure of T1-11 is *N* ^6^-(4-hydroxybenzyl)adenine riboside [21], which we subsequently confirmed by synthesis (Supplementary Fig. S3). T1-11 constitutes about 0.3% of the 75% MeOH fraction of GE. To the best of our knowledge, the clinical use of T1-11 has not previously been investigated. In PC12 cells, T1-11 protected against serum withdrawal-induced cell death in a dose-dependent manner (Fig. 1C). Staining with annexin V-FITC confirmed that T1-11 rescued serum-deprived PC12 cells from apoptosis (Fig. 1D).

### T1-11 is an agonist of the A_2A_R and an inhibitor of the adenosine transporter

We further characterized the pharmacological properties of T1-11 using radioligand binding assays. Of the 208 receptors/transporters tested, 10 µM T1-11 bound to only three molecules of the adenosinergic system including the A_2A_R, A_3_R, and an adenosine transporter - equilibrative nucleoside transporter 1 (ENT1; Table 1, Supplementary Table S1). Of these molecules, T1-11 bound to the A_3_R with the highest affinity (*K*_i_ = 0.1 µM, Table 1). However, at a concentration (100 µM) approximately 1000-fold higher than its *K*_i_ value, T1-11 induced less than 50% GTP binding compared to a well-characterized A_3_ agonist (2-Cl-IB-MECA, 3 µM, Supplementary Fig. S4). This low level of GTP binding is considered insufficient to trigger the G protein-dependent signaling of the A_3_R, suggesting that despite its strong binding affinity, T1-11 may not be a functional ligand for the A_3_R.

**Table 1:** Pharmacological properties of T1-11.

Target Molecules	IC_50_ (µM)	K_i_ (µM)	Function
A_1_ adenosine receptor	n.s.	n.s.	n.d.
A_2A_ adenosine receptor	4.66	2.62	agonist
A_2B_ adenosine receptor	n.s.	n.s.	n.d.
A_3_ adenosine receptor	0.11	0.10	n.s.
Adenosine transporter (ENT1)	1.57	0.54	inhibitor

**Figure 2:**
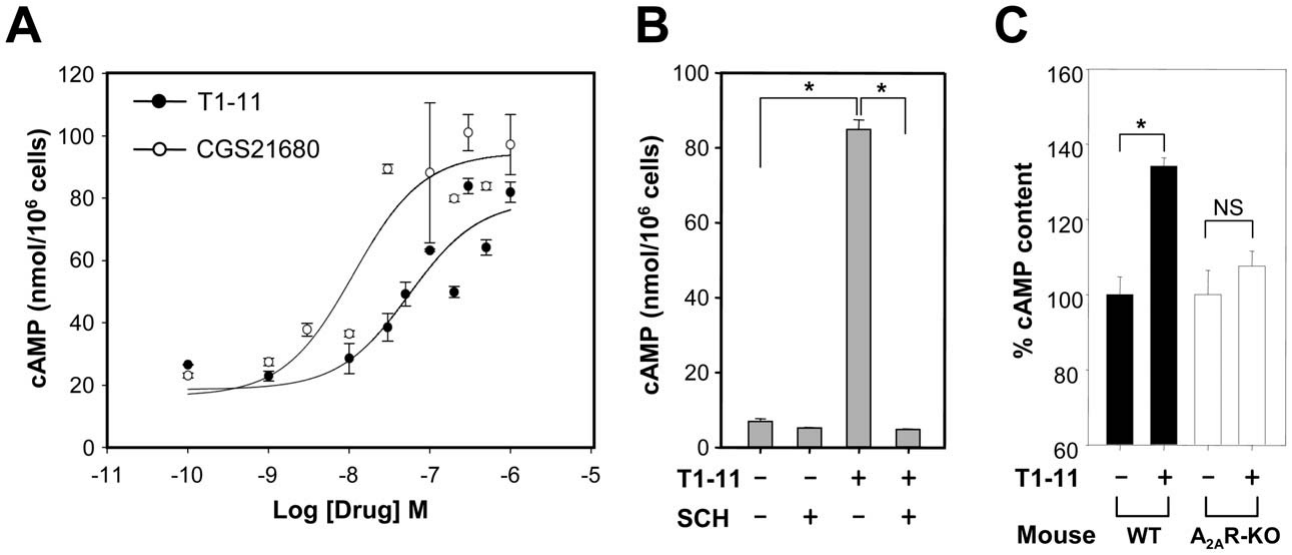
T1-11 is an agonist of the A_2A_R. (A) PC12 cells were treated with T1-11 (closed circles) and CGS21680 (open circles) at the indicated concentration for 20 min at room temperature (RT). (B) PC12 cells were stimulated with T1-11 (10 µM) in the absence or presence of an A_2A_R antagonist (SCH, 1 µM) for 20 min at RT. (C) Wildtype and A_2A_R knockout (KO) mice were intraperitoneally administrated with T1-11 (5 mg/kg body weight, *n* = 4) or vehicle for 60 min to measure the cAMP level in the brain.

**Figure 3:**
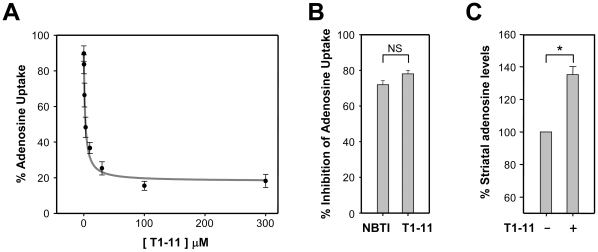
T1-11 inhibited the uptake of adenosine. (A) Adenosine uptake by PC12 cells was analyzed in the presence of T1-11 at the indicated concentration. (B) Adenosine uptake by PC12 cells was evaluated in the presence of T1-11 (30 µM) or NBTI (0.1 µM) as indicated for 10 min. (C) T1-11 (100 µM) was perfused throughout the dialysis probe. The collected perfusates were analyzed for striatal adenosine levels. Data points represent the mean ± s.e.m.. * *p*<0.05, compared to the basal level.

Binding properties of T1-11 toward four adenosine receptors (A_1_, A_2A_, A_2B_, and A_3_ receptors) and one adenosine transporter (ENT1) were conducted and characterized using standard binding protocols. T1-11 is an agonist of the A_2A_R to activate adenylyl cyclases and subsequently elevate cellular cAMP levels (Fig. 2). T1-11 also suppressed the uptake of adenosine (Fig. 3), and therefore was considered an inhibitor of ENT1. No significant binding of T1-11 toward the A_1_R or A_2B_R at 10 µM was found. Although T1-11 also bound to the A_3_R, it evoked no significant GTPγS binding at a concentration (10 µM) 1000-fold of its 1000-fold of its *K*_i_ value. n.d., not determined. n.s., not significant.

In contrast, T1-11 appeared to activate the A_2A_R (Table 1). We assessed the effect of two A_2A_R antagonists (ZM241385 and SCH58216) on T1-11's ability to prevent serum deprivation-induced death in PC12 cells and found that treatment with either antagonist blocked the effect of T1-11, indicating that T1-11's anti-apoptotic function is mediated at least in part by its role as a ligand for the A_2A_R (Fig. 1E). Treatment of PC12 cells with T1-11 dose-dependently elevated the cellular cAMP levels (Fig. 2A), and the EC_50_ value (∼2.2 µM) was similar to its binding property toward the A_2A_R (Table 1). The A_2A_R-selective antagonist (SCH58216, SCH) effectively blocked the T1-11-induced elevation in the cAMP level (Fig. 2B), further supporting the hypothesis that T1-11 activates a cAMP-dependent pathway by stimulating A_2A_R. Importantly, a single intraperitoneal injection of T1-11 (5 mg/kg body weight) increased cAMP levels in the brains of wildtype, but not A_2A_R knockout [25], mice (Fig. 2C). In addition, T1-11 was detected in the brain 30 min after the intraperitoneal injection (0.12±0.01 ng/g brain lysate, *n* = 4; mean ± s.e.m.). These data indicate that T1-11 enters the brain and elevates cAMP via activation of the A_2A_R *in vivo*.

T1-11 also bound to an adenosine transporter ENT1 (Table 1, *K*_i_ = 0.54 µM). T1-11 inhibited adenosine uptake by PC12 cells in a dose-dependent manner (Fig. 3A). The maximal inhibition of adenosine transport evoked by T1-11 at a concentration of 30 µM (∼55-fold its *K*_i_ value, Table 1) was similar to that caused by a well-characterized ENT1 inhibitor [nitrobenzylthioinosine, NBTI; 100 nM, 118-fold its *K*_i_ value (0.85 nM)] (Fig. 3B). Most importantly, introduction of T1-11 into the striatum of wildtype mice significantly enhanced the level of striatal adenosine as determined by microdialysis (Fig. 3C), demonstrating that T1-11 inhibited adenosine uptake *in vivo*. Thus, in addition to its ability to activate the A_2A_R, administration of T1-11 increases adenosinergic tone *in vivo*.

### T1-11 binds to the adenosine pockets of the A_2A_R and of the ENT1

**Figure 4:**
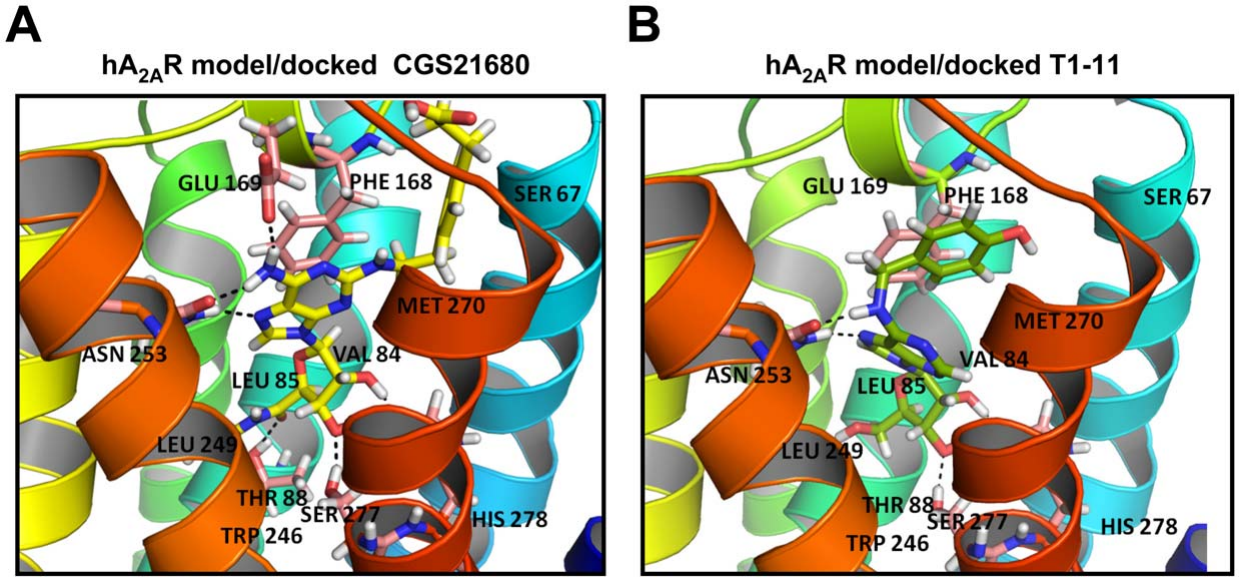
Interactions of the agonists with the ligand binding sites of the A_2A_R. (A) The binding pose of CGS21680 (a selective agonist) on the human A_2A_R, as predicted by combined homology modeling and docking analysis. The three-dimensional structure of the activated-state A_2A_R was constructed based on the inactive-state structure of the A_2A_R and the opsin structure. (B) Similar to (A), the binding pose of T1-11 on the human A_2A_R.

**Figure 5:**
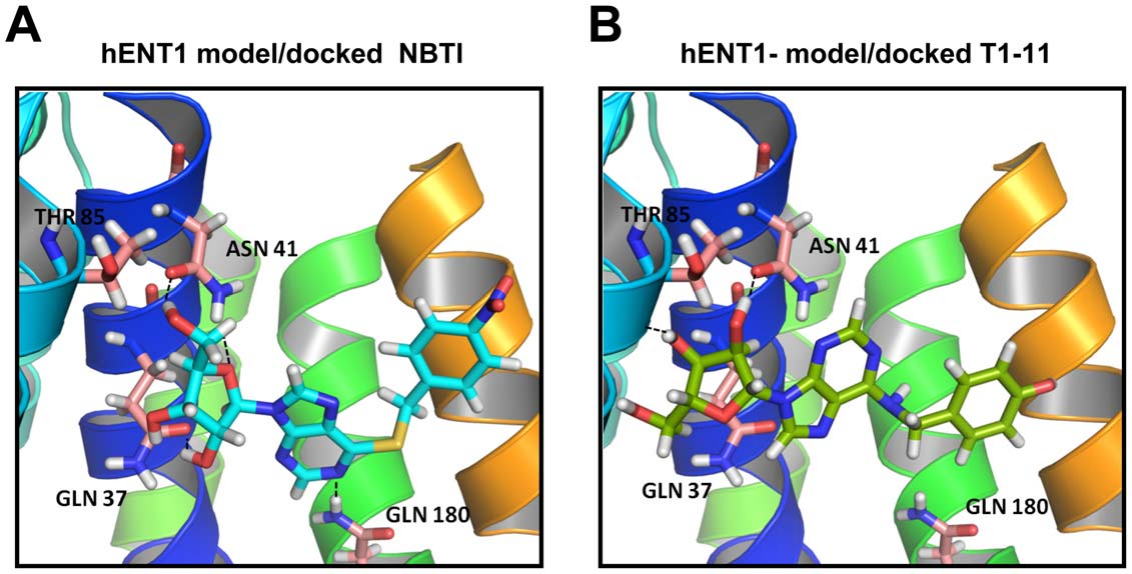
Interactions of inhibitors with ligand binding sites of ENT1. (A) The binding pose of NBTI (a selective inhibitor of ENT1) on human ENT1, as predicted using threading-based *ab inito* modeling of this transporter. The three-dimensional structure of ENT1 was constructed based on the lactose permease (GlpT) structure. (B) Similar to (A), the binding pose of T1-11 on human ENT1.

We next analyzed whether T1-11 fits into the ligand binding sites of activated A_2A_R (Fig. 4) and ENT1 (Fig. 5). From the newly resolved structure of human A_2A_R bound to the antagonist, ZM241385, in the inactive state [26] and previous mutagenesis experiments [17], [27], it was evident that the -NH_2_ interaction of the adenine core in CGS has the same binding interaction motifs as the antagonist, ZM241385 [26]. As shown in Figure 4A, the predicted binding of CGS to the structural model of the activated state of the human A_2A_R involves many hydrogen bonds with residues Thr88 [27], Asn253 [28], Glu169 [29], Ser277 [28], and His278 [30], which were identified by previous mutagenesis experiments. T1-11 docked to the same A_2A_R structural model, and was also involved in interactions with residues Asn253, Ser277, and His278, but with fewer hydrogen bonds (Fig. 4B). This analysis indicates that T1-11 fits into the ligand-binding site of the A_2A_R with a weaker affinity than CGS.

Because of the lack of a suitable structural template for homologous modeling of human ENT1 (hENT1), we conducted threading-based *ab inito* modeling of this transporter. The structural model of hENT1 resembles the structure of lactose permease (GlpT) [31], even though the number of transmembrane helices is different (11 for hENT1 vs. 12 for GlpT). This structure was further refined by a molecular-dynamics simulation in the fully solvated lipid bilayer, as detailed in “Materials and Methods”. Docking the well-known hENT1 inhibitor, NBTI, and T1-11 to the refined structure generated the binding modes depicted in Figure 5A and 5B, respectively. NBTI and T1-11 bound to the transporter in the substrate translocation channel with similar orientations at similar binding sites.

### Chronic treatment with T1-11 has beneficial effects on several major symptoms of HD in a transgenic mouse model of HD

**Figure 6:**
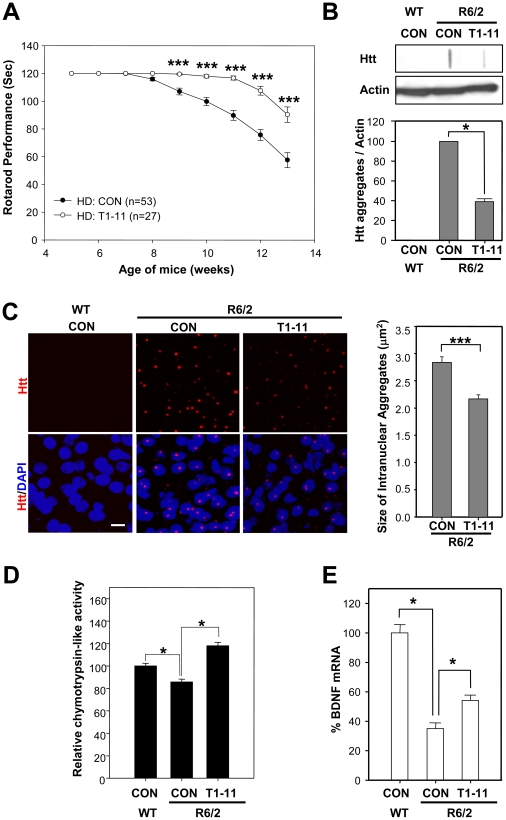
T1-11 exhibited beneficial effects in a mouse model of HD. R6/2 mice were given the vehicle (1% DMSO; CON, *n* = 53) or T1-11 (0.05 mg/ml, *n* = 27)-containing drinking water from the age of 7 weeks. (A) Rotarod performance was conducted as described in “Methods”. (B) Striatal lysates (50∼100 µg) collected from the indicated mice at the age of 12 weeks old were subjected to a filter retardation assay. The insoluble Htt aggregates retained on the filter were detected using an anti-Htt antibody (upper panel). The amount of protein in each corresponding lysate was independently assessed by Western blot analyses using an anti-actin antibody (middle panel). The relative aggregate formation was quantified by dividing the Htt signals in filter assays with those of the corresponding actin signals in Western blots (bottom panel). Data are presented as the mean ± s.e.m. values from three independent experiments. * *p*<0.05, versus R6/2 mice with no treatment (CON, Student's *t* test). (C) Brain sections of 12-week-old animals [vehicle (CON)-treated WT mice (*n* = 5), vehicle-treated R6/2 mice (*n* = 5), and T1-11-treated R6/2 mice (*n* = 5)] were stained with an anti-Htt antibody. Mutant Htt aggregates were visualized using Alexa Flour 568 (red). Nuclei were visualized using H33258 (blue). Representative pictures are shown. The scale bar is 10 µm. Data are presented as the mean ± SEM in each group. *** *p*<0.001, versus R6/2 mice treated with vehicle (CON, Student's *t* test). (D) The chymotrypsin-like activity of proteasomes in striatal synaptosomes of the indicated 12-week-old mice (*n* = 3) was assessed as described in “Methods”. (E) Cortical tissues (n = 3) were collected to determine the transcript level of BDNF using a quantitative RT-PCR technique. The expression levels of BDNF were normalized to that of GAPDH. * P<0.05, versus R6/2 mice with no treatment (CON, Student's *t* test).

As the A_2A_R and ENT1 are located in the striatum and have been implicated in striatal function [32], we hypothesized that chronic treatment with T1-11 would modulate the progression of HD. We first tested the effect of T1-11 in a transgenic mouse model (R6/2) of HD in which A_2A_R agonists have beneficial effects [12], [13]. The addition of T1-11 (0.05 mg/ml) to the drinking water of mice from the age of 7 weeks counteracted the progressive deterioration in motor coordination as assessed by rotarod performance (Fig. 6A). The mean survival times of control and T1-11-treated R6/2 mice were 99.0±2.1 d (*n* = 22) and 103.3±3.9 d (*n* = 11), respectively (Supplementary Fig. S5A). Using *in vivo* 3D MRI imaging, we found that T1-11 slightly ameliorated the brain atrophy of R6/2 mice but the improvement did not reach statistic significance (Supplementary Fig. S5B). Importantly, chronic treatment with T1-11 markedly reduced the formation of striatal Htt aggregates, a hallmark of HD, as assessed by filtered retardation assays (Fig. 6B) and immunofluorescence analyses (Fig. 6C). We recently reported that activation of the A_2A_R enhances mHtt-induced suppression of proteasome activity via a PKA-dependent pathway in the liver [12]. Therefore, we determined whether T1-11 decreases aggregate formation by elevating proteasome activity. As shown in Figure 6D, chymotrypsin-like activity in the striatal synaptosome fractions was lower in HD mice than in wildtype mice. Chronic treatment with T1-11 significantly enhanced chymotrypsin-like activity in the HD striatum (Fig. 6D). We also assessed whether T1-11 modulates other changes in brains of R6/2 mice. A previous study showed that the level of brain derived neurotrophic factor (BDNF) was decreased in the brains of HD mice [33]. Consistent with the beneficial effects of T1-11 on motor coordination, we found that T1-11-treated R6/2 mice contained more cortical BDNF than R6/2 mice that received no treatment (Fig. 6E). These results demonstrate the therapeutic potential of T1-11 for treating HD.

## Discussion

We identified a novel adenosine analogue, T1-11, that possesses a dual function - both activating adenosine receptors and blocking the adenosine transporter ENT-1. Our data suggest that by simultaneously activating the A_2A_R and inhibiting adenosine uptake, T1-11 produces beneficial effects on HD by selectively elevating the adenosinergic tone of the brain, a novel protective mechanism.

Adenosine is an important endogenous neuroprotective substance and is a metabolite of many biosynthetic pathways. The endogenous adenosine level is known to be associated with the status of energy homeostasis in the brain [5]. Certain psychopharmacological agents (e.g., caffeine and ethanol) function by modulating the endogenous adenosine tone of the brain [34]. Our finding that T1-11-mediated elevation of adenosine tone had beneficial effects in R6/2 mice is consistent with a previous report showing that an increase in the adenosine tone of the brain exerts a protective effect on cerebral ischemia [35]. Modulation of the adenosine tone by pharmacological means may be useful in developing therapies for neurodegenerative diseases and/or traumas of the CNS. Several interesting adenosine drugs have been developed. For example, propentofylline, a weak inhibitor of three adenosine receptors (with a preference for the A_1_R) and adenosine transporters, can be used to treat dementia and ischemic brain damage [36]. The action of propentofylline is intriguing and complex as it indirectly enhances functions of adenosine receptors via inhibition of adenosine transporters which elevate extracellular adenosine concentrations, and directly suppresses adenosine receptors, limiting its former action [36]. Although the binding affinities of T1-11 were not as strong as those of the best adenosine drugs currently available (Table 1, Figs. 2, 3, 6), the dual functions of these compounds in activating adenosine receptors and inhibiting adenosine transporters is likely to enable T1-11 to effectively activate the adenosinergic system in synapses where both adenosine receptors and transporters are located.

Despite the significant interest in A_2A_R-related drugs for HD, earlier studies using different mouse models of HD showed complex and even conflicting conclusions on the neuroprotective *versus* neurodegenerative roles of the A_2A_R in HD. We earlier published a review in which we evaluated whether the A_2A_R is a feasible drug target for HD, and concluded that more studies are needed to clarify the application of A_2A_R drugs to HD [37]. Because stimulation of the A_2A_R triggers glutamate release, it was proposed that the presynaptic A_2A_R on the glutminergic terminals would be harmful, while that on the postsynaptic GABAergic terminals would be protective [10], [15], [16]. On the contrary, the A_2A_R is closely linked to BDNF which is markedly impaired in HD [7], [33], [38], [39]. Activation of the A_2A_R enhances the signal of BDNF by facilitating localization of its receptor (TrkB) in lipid rafts through a cAMP/PKA-dependent pathway, transactivates TrkB, and increases the synthesis of TrkB [40], [41], [42]. Stimulation of the A_2A_R also facilitates the functions of other neurotrophic factors [such as the glial cell line-derived neurotrophic factor (GDNF) and fibroblast growth factor (FGF)]. These findings are important because supplementation with BDNF, GDNF, or FGF in HD mice all led to beneficial effects on disease progression [43], [44]. Small molecules which activate the A_2A_R thus might provide a unique means to elicit trophic responses in HD. It is important to note that the expression and signaling of the A_2A_R as well as other receptors (e.g., D2 dopamine receptor and metabotropic glutamate receptor) are altered in both HD mice and patients [13], [45]–[49]. The roles of the A_2A_R revealed by studies conducted in wildtype animals therefore need to be re-evaluated in genetic models of HD. Indeed, studies from several laboratories showed that activation of the A_2A_R produces opposite effects in the striatum of WT and HD (R6/2) mice [11], [50]. In R6/2 mice, treatment with an A_2A_R antagonist (SCH58261) for 1 week worsened motor coordination [51]. Genetic removal of the A_2A_R in another HD mouse model (N171-82Q) also exacerbated motor degeneration and shortened the lifespan [14]. However, the abovementioned 1-week treatment with SCH58261 in the presymptomatic stage of R6/2 mice was associated with a reduction in NMDA-induced toxicity, indicating a potential protective effect [51]. Because the glutamate release was dynamically altered from an enhancement at the presymptomatic stage to a decrease at the symptomatic stage during disease progression of HD mice [49], and since local glutamate level was recently shown to dictate the effect of A_2A_R on neuronal death in an animal model of traumatic brain injury [52], it is possible that the stage of disease progression might contribute to the complex role of A_2A_R in HD. Further investigations using both pharmacological and genetic approaches are necessary to verify whether early and/or chronic blockage of the A_2A_R is detrimental during HD progression. Conversely, activation of the A_2A_R using CGS provides beneficial effects on several major HD symptoms (including brain atrophy, striatal aggregates, and deteriorated motor coordination) in R6/2 mice [12], [13]. In line with our studies, Cepeda and colleagues also demonstrated that CGS ameliorates the corticostriatal synaptic disconnection in R6/2 mice [11]. Agonists of the A_2A_R therefore might be used to treat HD. Unfortunately, full A_2A_ agonists (e.g., CGS) have unfavorable acute side effects, including low food intake, sedation/drowsiness, an increased heart rate, and systematic hypotension [17], [53], [54]. A modest agonist such as T1-11 may have fewer side effects in peripheral tissues and may thus be superior to full A_2A_ agonists for therapeutic uses such as those of other classes of adenosine drugs [55]. We tested this hypothesis by treating R6/2 mice with T1-11 using a subcutaneous Alzet minipump for 48 h. At a dose that improved motor deterioration, T1-11 did not change the blood pressure of R6/2 mice (Supplementary Materials, Figure S6). The rapid entry of T1-11 into the brain suggests that it may have great potential for treating brain diseases. Collectively, T1-11 possesses key features of an ideal A_2A_ drug for HD. In the present study, T1-11 was delivered via the drinking water because it is one of the most common routes for drug delivery in human patients and it causes much less stress on mice than intraperitoneal injection or oral gavage. Nonetheless, such mode of drug delivery did not permit an accurate assessment of the actual doses taken by the animals. In addition, HD mice at late stage of the disease drink less water because of impaired motor function, and thus receive less T1-11. Such reduced intake of T1-11 by mice with late stage HD might compromise the beneficial effect of T1-11 and account for the inability of T1-11 to rescue certain symptoms of HD (e.g., shorten lifespan and brain atrophy, Supplementary Figure S5). For potential clinical application of T1-11 in the future, it is critical to further optimize its effective dose, formulation, and administration protocol to maximize its beneficial effect.

The protective effects of the GE extract and T1-11 are consistent with a previously implied neuroprotective effect of GE [18], [19]. By targeting multiple components in the adenosinergic system, T1-11 is expected to elevate the adenosine tone and is a potential candidate for treating HD. In addition to the role of the A_2A_R as discussed above, the functions of other adenosine receptors (particularly, the A_1_R and A_3_R) in HD also warrant further studies. In an earlier report, an A_1_R-specific agonist (adenosine amine congener) showed neuroprotective effects in a rat HD model created by the systemic administration of 3-nitropropionic acid which caused striatal lesions [56]. The functions of the A_3_R have not been evaluated in HD before. Nonetheless, agonists of the A_3_R provide neuroprotective effects against subarachnoid hemorrhage-induced brain damage [57]. The contributions of the A_1_R and A_3_R to the beneficial effects of T1-11 on HD mice require further experimental evaluation.

The concept of a small molecule that targets multiple components in the same regulatory system to optimize its function at a specific location (such as synapses) is a novel strategy for developing therapeutic methods for HD, for which there is currently no effective treatment [58]. This approach is particularly critical for the design of neurotransmitter-based drugs for CNS diseases in which side effects from peripheral tissues are a major obstacle. Dual-action drugs have recently attracted much attention [59]. A drug (tapentadol) recently approved by the US FDA, also a dual-action molecule, acts on molecules of two different neurotransmitter systems (a μ-opioid receptor agonist and a norepinephrine transporter) [60]. Another interesting example is 8-(3-chlorostyryl)caffeine, a well-characterized A_2A_-selective antagonist which ameliorates MPTP neurotoxicity by simultaneously inhibiting monoamine oxidase and the A_2A_R [61]. A similar design as for dual-function drugs may be applicable to other neurotransmitter systems (e.g., dopamine receptor/transporter and serotonin receptor/transporter) and may facilitate the development of new drugs for other neurodegenerative diseases.

## Materials and Methods

### Preparation of the GE extract and T1-11

The rhizome of *G. elata* (GE) was purchased from a local herbal store in Taipei. Slices of GE were extracted at 60°C using 80% ethanol/H_2_O overnight (3 times). The crude extract was concentrated using a vacuum rotary evaporator (Büchi) under reduced pressure. The dried sample (about 15% yield based on the dried herbal weight) was subjected to Diaion HP-20 column chromatography using elution from a H_2_O/MeOH gradient. Fractions were examined for their abilities to prevent apoptosis induced by serum withdrawal in PC12 cells. The active fractions of 50%∼75% MeOH/H_2_O were combined and purified on a Sephadex LH-20 column by repeated elution with MeOH to give T1-11. High-pressure liquid chromatography was performed on a Merck RP-18e (250×4.6 mm) column using a mobile phase gradient from 70% to 40% H_2_O/MeOH for 40 min and from 40% to 20% H_2_O/MeOH for 5 min at a flow rate of 0.8 ml/min. A UV 270-nm detector was used to monitor the chemical profiles of different batches.

### Synthesis of T1-11

Compound T1-11 was synthesized in a high yield by the substitution reaction of 6-chloropurine ribonucleoside with 4-hydroxybenzylamine (as hydrochloric acid) in the presence of a base diisopropylethylamine [62], [63]. Because the hydrochloric salt of 4-hydroxybenzylamine is not commercially available, it was prepared by hydrogenation of the corresponding 4-hydroxybenzaldehyde oxime.

### Cell culture

PC12 cells purchased from ATCC (Manassas, VA, USA) were maintained in Dulbecco's modified Eagle's medium (DMEM) supplemented with 10% horse serum and 5% fetal bovine serum and incubated in a CO_2_ incubator (5%) at 37°C.

### MTT metabolism assay

Survival was assessed by the 3-(4,5-dimethylthiazol-2-yl)-2,5-diphenyltetrazolium bromide (MTT) metabolism assay as described elsewhere [64], [65]. In brief, cells grown on 150-mm plates were washed three times with PBS and resuspended in DMEM. Suspended cells (1×10^4^ cells) were plated on 96-well plates and treated with or without the indicated reagent. After incubation for 24 h, MTT (0.5 mg/ml) was added to the medium and incubated for 3 h. After discarding the medium, DMSO (100 µl) was then applied to the well to dissolve the formazan crystals derived from the mitochondrial cleavage of the tetrazolium ring by live cells. The absorbance at 570/630 nm in each well was measured on a micro-enzyme-linked immunosorbent assay reader.

### Annexin V-FITC staining

An annexin V (FITC-conjugated) apoptosis kit (K101-400; BioVision, Mountain View, CA, USA) was used to analyze apoptotic cells. The experimental protocol followed the manufacturer's instructions and a previous article [66]. In brief, after treatment with serum, serum-free medium, or serum-free medium plus T1-11 of the indicated concentration for 24 h, cells growing on 12-well plates at (3∼4)×10^5^ cells/well were loaded with 0.5 ml binding buffer and 5 µl annexin V-FITC. After incubation for 5 min in the dark, cells were washed once with 1 ml of culture medium (without phenol red) for fluorescent imaging analyses (Axiovert-200M, Carl Zeiss, Gôttingen, Germany) or a flow cytometric analysis (Beckton Dickinson, Franklin Lakes, NJ, USA). Median values of the FITC fluorescent intensities were determined using an FL-1 channel (488/530Ex/Em nm). Five thousand live cells were analyzed per sample.

### Radioligand binding assays

Radoligand binding assays were performed by MDS Pharma Services Taiwan (Taipei, Taiwan) using standard binding protocols. For the binding assay of the A_2A_R [67], membrane proteins collected from HEK293 cells overexpressing the human A_2A_R were incubated in reaction buffer [50 mM Tris-HCl (pH 7.4), 10 mM MgCl_2_, 1 mM EDTA, and 2 U/mL adenosine deaminase] containing ^3^H-CGS21680 (50 nM) for 90 min at 25°C. Nonspecific binding was assessed in the presence of 50 µM adenosine- 5′-*N*-ethylcarboxamide. To measure the binding affinity of T1-11 to the A_3_R [68], [69], membrane proteins collected from CHO-K1 cells overexpressing the human A_3_R were incubated with ^3^H-AB-MECA (0.5 nM) for 60 min at 25°C in a reaction buffer containing 25 mM HEPES (pH 7.4), 5 mM MgC_2_, 1 mM CaCl., and 0.1% bovine serum albumin. Nonspecific binding was assessed in the presence of 1 µM IB-MECA (Tocris Bioscience, Ellisville, MS, USA). Binding assays for adenosine transporters were conducted as described earlier ([70]. Membrane fractions collected from the cerebral cortex of Duncan Hartley derived guinea pigs were incubated with ^3^H-labeled 6-[(4-nitrobenzyl)thio]-9-β-D-ribofuranosylpurine (NBTI, 0.5 nM) for 30 min at 25°C in an incubation buffer containing 50 mM Tris-HCl (pH 7.4). Nonspecific binding was assessed in the presence of 5 µM NBTI, an effective inhibitor of equilibrative nucleoside transporters. Note that NBTI is a high-affinity inhibitor of ENT1, and inhibits only human (h)ENT1 at 0.5 nM [71]. Reactions were terminated by filtration over GF/B glass fibers and washing with the corresponding reaction buffer.

### cAMP assay

PC12 cells were plated at the density of 5×10^5^ cells/well (on 12-well plates) and incubated with the indicated reagent(s) for the desired period of time. Cells were washed twice with ice-cold Locke's solution (150 mM NaCl, 5.6 mM KCl, 5 mM glucose, 1 mM MgCl_2_, and 10 mM HEPES, adjusted to pH 7.4). Cellular cAMP was extracted by adding 0.3 ml of 0.1 M HCl to each well and incubating this for 10 min on ice. The cAMP content was assayed using the ^125^I-cAMP assay system (GE Healthcare, Little Chalfont, Buckinghamshire, UK).

C57BL6 mice (8 weeks old) were intraperitoneally (i.p.) administrated with T1-11 (5 mg/kg body weight, *n* = 4) or vehicle for 60 min. Brain tissues were carefully removed, and homogenized in 1 ml of assay buffer (25 mM Tris (pH 8), 1 mM EGTA, 1 mM MgCl_2_, 40 mM leupeptin, 100 mM PMSF, 10 nM okadaic acid, 1× EDTA free proteinase inhibitor cocktail (Roche), 0.5 mM IBMX, and 20 mM papaverine). Trichloroacetic acid (6%, final concentration) was then added to the lysate to precipitate proteins. The cAMP level in the supernatant was analyzed as previously described.

### GTPγS binding assay

The GTPγS binding assay was conducted by MDS Pharma Services Taiwan using a previously described protocol [55] with slight modifications. In brief, membrane proteins collected from Chinese hamster ovary (CHO-K1) cells expressing the human A_3_R (5∼10 µg per reaction) were incubated with the indicated drug and ^35^S-GTPγS (0.1 nM) in a total volume of 500 µl for 30 min at 30°C. The reaction buffer was composed of 20 mM HEPES (pH 7.4), 100 mM NaCl, 10 mM MgCl_2_, 1 mM DTT, and 1 mM EDTA. The reaction was terminated by filtration over GF/B glass fibers and washed with the same reaction buffer. Relative GTPγS binding was defined as the percentage of ^35^S-GTPγS binding when compared to that of a selective agonist of the A_3_R (2-Cl-IB-MECA, 3 µM) under the same binding conditions.

### Adenosine transport

For adenosine uptake of PC12, cells were seeded approximately 16 h before each uptake assay at ∼2×10^5^ cells per well in 24-well plates coated with poly-L-lysine. To perform adenosine uptake assays, cells were washed with Krebs Ringer-Henseleit buffer [125 mM NaCl, 4.8 mM KCl, 1.3 mM CaCl_2_, 1.2 mM MgSO_4_, 1.2 mM KH_2_PO_4_, 5.6 mM glucose, 10 µM pargyline, and 10 mM HEPES (pH 7.2)] and incubated in the same buffer for 10 min at 37°C in the presence or absence of the indicated reagent(s). Adenosine uptake was initiated by adding [^3^H]adenosine at the indicated concentration (0.5 µCi/mmol) at 37°C. At the end of the incubation, cells were placed on ice, washed twice with ice-cold Krebs Ringer-Henseleit buffer to remove free [^3^H]adenosine, lysed with 1% Triton X-100, and added to scintillation vials to count the radioactivity. Non-specific uptake was determined as the uptake performed in the presence of 100 µM adenosine, and was subtracted from the total adenosine uptake.

### *In vivo* brain dialysis and adenosine measurement

The level of adenosine in the brain was determined using microdialysis as previously described [72], [73]. In brief, concentrated dialysis probes with 4-mm dialysis membranes (CMA, Stockholm, Sweden) were used to monitor the extracellular adenosine in the striatum of rats. After inducing anesthesia with chloral hydrate (400 mg/ml, IP), rats were implanted with the probe, and the coordinates for implantation were AP +1.0 mm, LM +2.8 mm, and VD −6.5 mm. Ringer's solution (140 mM NaCl, 1.2 mM CaCl_2_, 3.0 mM KCl, 1.0 mM MgCl_2_, and 0.04 mM ascorbic acid) was continuously perfused (0.5 µl/min) via probes throughout the experiments. After implantation and perfusion for 1.5 h, the perfusate was collected for 1 h as a baseline and then Ringer's solution containing T1-11 (100 µM) was perfused for another 1 h. The perfusate was analyzed by high-performance liquid chromatography (HPLC; Agilent 1100 series, Germany) coupled with a photo diode array detector (Agilent G1315B,) at 260 nm. Separations were obtained with a reversed-phase column (Cosmosil 5C18-AR-II, 250×4.6 mm, Kyoto, Japan) eluted at a flow rate of 1.0 ml/min with a linear solvent gradient elution system composed of eluents A and B (A: 0.0085% H_3_PO_4_ in H_2_O; B: 100% acetonitrile) according to the following profile: 0∼15 min, 100%∼90% A, 0%∼10% B.

### Animals and drug administration

Male R6/2 mice [74] and littermate controls were originally obtained from Jackson Laboratories (Bar Harbor, ME, USA), and mated to female control mice (B6CBAFI/J). Offspring were identified by a polymerase chain reaction (PCR) genotyping technique of genomic DNA extracted from tail tissues using primers located in the transgene (5′-CCGCTCAGGTTCTGCTTTTA-3′ and 5′-GGCTGAGGAAGCTG- AGGAG-3′) to ensure that the number of CAG repeats remained approximately 150. In total, 211 R6/2 transgenic mice were used in this study. Animals were housed at the Institute of Biomedical Sciences Animal Care Facility under a 12-h light/dark cycle. Body weights of mice were recorded once daily. Animal experiments were performed under protocols approved by the Academia Sinica Institutional Animal Care and Utilization Committee, Taipei, Taiwan.

### Rotarod performance

Motor coordination was assessed using a rotarod apparatus (UGO BASILE, Comerio, Italy) at a constant speed (12 rpm) over the period of 2 min [75]. All mice were trained for 2 days at the age of 4 weeks to allow them to become acquainted with the rotarod apparatus. Animals were then tested three times per week from the ages of 4∼12 weeks. For each test, animals were placed in the apparatus before initiation of rotation. Latency to falling was automatically recorded. Each mouse was given three trials for a maximum of 2 min for each trial.

### Hemodynamic Examination

Heart rates and blood pressure of conscious and anesthetized mice were measured using a blood pressure monitor (model MK-2000; Muromachi Kikai, Tokyo, Japan) between 10 AM to 6 PM. Values of 12–18 successful readings per mouse were used to determine the blood pressure.

### Filter retardation assay

SDS-insoluble mutant Htt aggregates were detected and quantified as described [76]. A filter retardation assay was performed as described previously [2]. Blots were blocked with 5% skim milk in phosphate-buffered saline (PBS) and incubated with an anti-Htt antibody (EM48, 1∶500; Chemicon International, Temecula, CA, USA) at 4°C overnight followed by the corresponding secondary antibody for 1 h at room temperature. Immunoreactive bands were detected by enhanced chemiluminescence (Pierce) and recorded using Kodak XAR-5 film.

### Immunohistochemistry and quantitation

Coronal serial sections (20 µm) containing the striatum (interaural 5.34 mm/bregma 1.54 mm to interaural 3.7 mm/bregma −0.1 mm) were immunohistochemically stained as described previously [77]. Brain sections were blocked with normal goat serum and incubated overnight with an anti-Htt antibody (EM48, 1∶500) at 4°C, followed by a 2 h-incubation with a goat anti-mouse IgG conjugated to Alexa Fluor® 568 at room temperature. The nuclei were stained with Hoechst 33258. The patterns of immunostaining were analyzed with a laser confocal microscope (LSM510, Carl Zeiss MicroImaging Inc, Germany). Five different brain sections of each animal were analyzed. At least 2000 cells from each animal were used to quantify the sizes of mHtt aggregates using ImageJ software (http://rsbweb.nih.gov/ij/; Research Services Branch of the National Institute of Mental Health, Bethesda, MD, USA).

### Proteasome activity assay

The chymotrypsin-like activity of the proteasome was determined using a specific proteasome substrate [succinyl (suc)-Leu-Leu-Val-Tyr-7-amino-4-methyl coumarin (AMC)] (Sigma-Aldrich, St Louis, MO, USA) as described earlier [12]. In brief, the synaptosome-enriched fraction (10 µg) were incubated with the substrate (40 µM) in 100 µl of proteasome assay buffer [0.05 M Tris-HCl (pH 8.0), 0.5 mM EDTA, 1 mM ATP, and 1 mM DTT] at 37°C for 60 min where the relationship between the incubation time and product formation remained linear. The fluorescence of the released AMC was detected using a Fluorescence Microplate Reader System (Device, Sunnyvale, CA, USA) at 380-nm excitation and 460-nm emission wavelengths.

### Western blot assays

Equal amounts of protein were separated by sodium dodecylsulfate polyacrylamide gel electrophoresis (SDS-PAGE) using 10% polyacrylamide gels according to the method of Laemmli [78]. The resolved proteins were electroblotted onto Immobilon polyvinylidene difluoride membranes (Millipore, Bedford, MA, USA). Membranes were blocked with 5% skim milk in PBS and incubated with an anti-actin antibody (1∶2500; Chemicon International) at 4°C overnight followed by the corresponding secondary antibody for 1 h at room temperature. Immunoreactive bands were detected by enhanced chemiluminescence (Pierce) and recorded using Kodak XAR-5 film.

### RNA isolation and quantitative real-time PCR

Total RNA was isolated from the cortex of the indicated mice using the TriReagent kit (Molecular Research Center, Cincinnati, OH, USA), treated with RNase-free DNase (RQ1; Promega) to remove potential contamination by genomic DNA, and transcribed into complementary (c)DNA using Superscript* II reverse transcriptase. A real-time quantitative PCR was performed using a TaqMan kit (PE Applied Biosystems, Foster City, CA, USA) on a TaqMan ABI 7700 Sequence Detection System (PE Applied Biosystems) using heat-activated TaqDNA polymerase (Amplitaq Gold; PE Applied Biosystems). The PCR mixtures were incubated at 50°C for 2 min and 95°C for 10 min, and then 40 PCR cycles were conducted (95°C for 15 s and 65°C for 1 min). The sequences of primers are listed below: for BDNF (the target gene), 5′-GGCTTCACAGGAGACATCAG-3′ and 5′-CAGAACCAGAACGAACAGAAAC-3′; and for GAPDH (the reference gene), 5′TATCCGTTGTGGATCTGACAT-3′ and 5′-ACAACCTGGTCCTCAGTGTA-3′. Independent reverse-transcription PCRs were performed using the same cDNA for both the indicated target gene and reference gene (GAPDH). A melting curve was created at the end of the PCR cycle to confirm that a single product had been amplified. Data were analyzed using ABI 7700 operating software to determine the threshold cycle (CT) above the background for each reaction. The relative transcript amount of the target gene, which was calculated using standard curves of serial RNA dilutions, was normalized to that of GAPDH of the same RNA.

### Structure modeling of the activated human adenosine A_2A_ receptor

Multiple sequence alignment was performed with ClustalW [79] for bovine rhodopsin, and the human adenosine A_1_, A_2A_, A_2B_, and A_3_ receptors, where the BLOSUM scoring matrix, a gap open penalty of 10, and a gap extension penalty of 0.05 were used. Sequences were retrieved from Swiss-Prot [80]. To model the active state of the human adenosine A_2A_R (residues 1∼310), residues 1∼172 (except for the unresolved residues, 1 and 2, and 148∼156) were taken from the recently solved inactivated structure (PDB ID: 3EML) [26], because it was found that these residues probably remained intact during the transition between the active and inactive states [81]. The extracellular loop 2, which should contribute to the binding of ligands to the receptor, is therefore based on the inactive structure of the A_2A_R. The unsolved residues, 1 and 2 and 148∼156 were patched with the program loopy [82]. TM5, TM6, TM7, and helix 8, i.e., residues 175∼310, were modeled based on the opsin structure [81]. MODELLER9v5 [83] was used for the chimerical modeling, and 100 models were generated. Finally, the model with the lowest DOPE energy [84] was selected. The hydrogen atoms were added by the PDB2PQR web server [85] at pH 7, but the N_δ_ atom of His278 was manually assigned to be protonated and the N_ε_ atom was not protonated, because the binding mode of this protonation assignment would be in better agreement with the mutation experiment. During the revision of the manuscript, the structure of A_2A_R bound with the agonist UK-432097 has been published [86]. We compared the model we constructed in this work with the newly released structure and found that these two structures are very similar, especially at the binding pocket of the receptor.

### Structure modeling of hENT1

The sequence of hENT1 was retrieved from Swiss-Port [80]. The initial models of hENT1 were constructed by iTASSER [87], which is the iterative implementation of the Threading ASSEmbly Refinement (TASSER) program [88]. One model was selected based on the spatial distribution of the transmembrane regions annotated by Swiss-Prot, to see whether the all the transmembrane helices can be packed into the hydrophobic slab of a membrane. All other models generated by iTASSER failed to meet this criterion. This hENT1 model was inserted into the POPC bilayer equilibrated in a previous study [89], where all the lipids and water molecules having van der Waals contacts with the transporter were removed. The system was energy-minimized by the steepest descent method for 500 cycles, with all transporter atoms restrained. The system was then equilibrated by conventional molecular dynamics at 300 K and 1 bar for 1 ns. The self-guided molecular dynamics simulation [90] was then conducted for 40 ns to refine the hENT1 structure. The lipid force field parameters were adopted from a previous study [89], and the AMBER parm99SB force field [91] was used for the transporter. The sander module of AMBER 9 [92] was employed for the simulations.

### Docking protocols

The partial charges of atoms on the ligand and the receptor molecules were determined by the Gasteiger method [93], aided by AutoDockTools. The number of chromosomes was set to 100, and the number of generations was set to 5000. The Solis-Wet local search iteration was set to 600. The binding pose of CGS21680 was predicted with the program AutoDock 4 [94].

## Supporting Information

Figure S1 **^1^H NMR spectrum of T1-11 (DMSO-** ***d*** **_6_, 400 MHz).**
(TIF)

Figure S2 **^13^C NMR spectrum of T1-11 (DMSO-** ***d*** **_6_, 100 MHz).**
(TIF)

Figure S3 **HPLC diagram of a synthetic sample of T1-11.**
(TIF)

Figure S4 **T1-11 binds to the A_3_ adenosine receptor (A_3_R) without evoking a significant binding of GTP.** Membrane fractions collected from CHO-K1 cells expressing the human A_3_R were incubated with T1-11 at the indicated concentration and ^35^S-GTPγS (0.1 nM) for 30 min at 30°C. Relative GTPγS binding was defined as the percentage of ^35^S-GTPγS binding when compared with a selective agonist of the A_3_R (2-Cl-IB-MECA, 3 µM).
(TIF)

Figure S5 **Effect of T1-11 on the shorten lifespan and enlarged ventricle of R6/2 mice.** Animals were given the vehicle (1% DMSO; CON) or T1-11 (0.05 mg/ml)-containing drinking water from the age of 7 weeks. (A) Survival was assessed. Specific comparison to R6/2 mice treated with the vehicle (*p* = 0.306; Mantel-Cox test). (B) Five weeks after T1-11 treatment, 3D-lMRI was performed to determine the ventricle-to-brain ratio of the indicated animals as described. * *p*<0.05.
(TIF)

Figure S6 **Treatment of R6/2 mice with T1-11 did not affect their heart rate and blood pressure.** (A) T1-11 (125 µg/mouse/day) or vehicle (CON) was administrated subcutaneously to the indicated mice of 7 weeks old using ALZET osmotic minipumps for 6 weeks. Rotarod performance was assessed. (B, C) T1-11 (125 µg/mouse/day) was administrated subcutaneously to the indicated mice of 9 weeks old using ALZET osmotic minipumps for 48 h. Heart rate (B) and blood pressure (C) were determined by a tail-cuff method. * *p*<0.05. *** *p*<0.005.
(TIF)

Table S1 Binding properties of T1-11 toward 208 proteins.
(PDF)
