# Supplementary figures and images for: A New Drug Design Targeting the Adenosinergic System for Huntington's Disease
Source: PLoS One. 2011 Jun 21;6(6):e20934. doi: 10.1371/journal.pone.0020934 (PMC3119665; doi:10.1371/journal.pone.0020934)

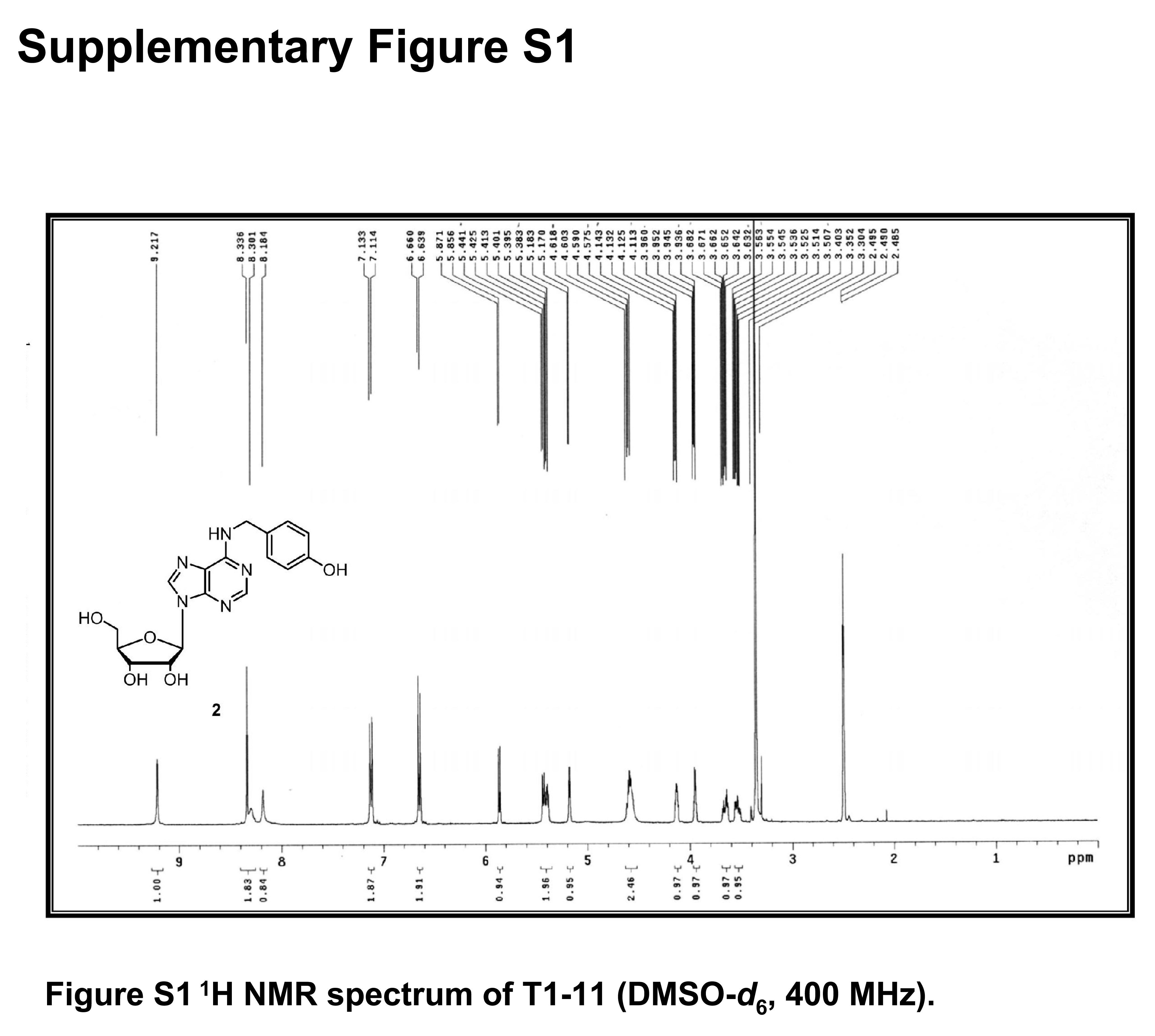

Supplement: Figure S1 — 1H NMR spectrum of T1-11 (DMSO- d 6, 400 MHz). (TIF) [file pone.0020934.s001.tif]

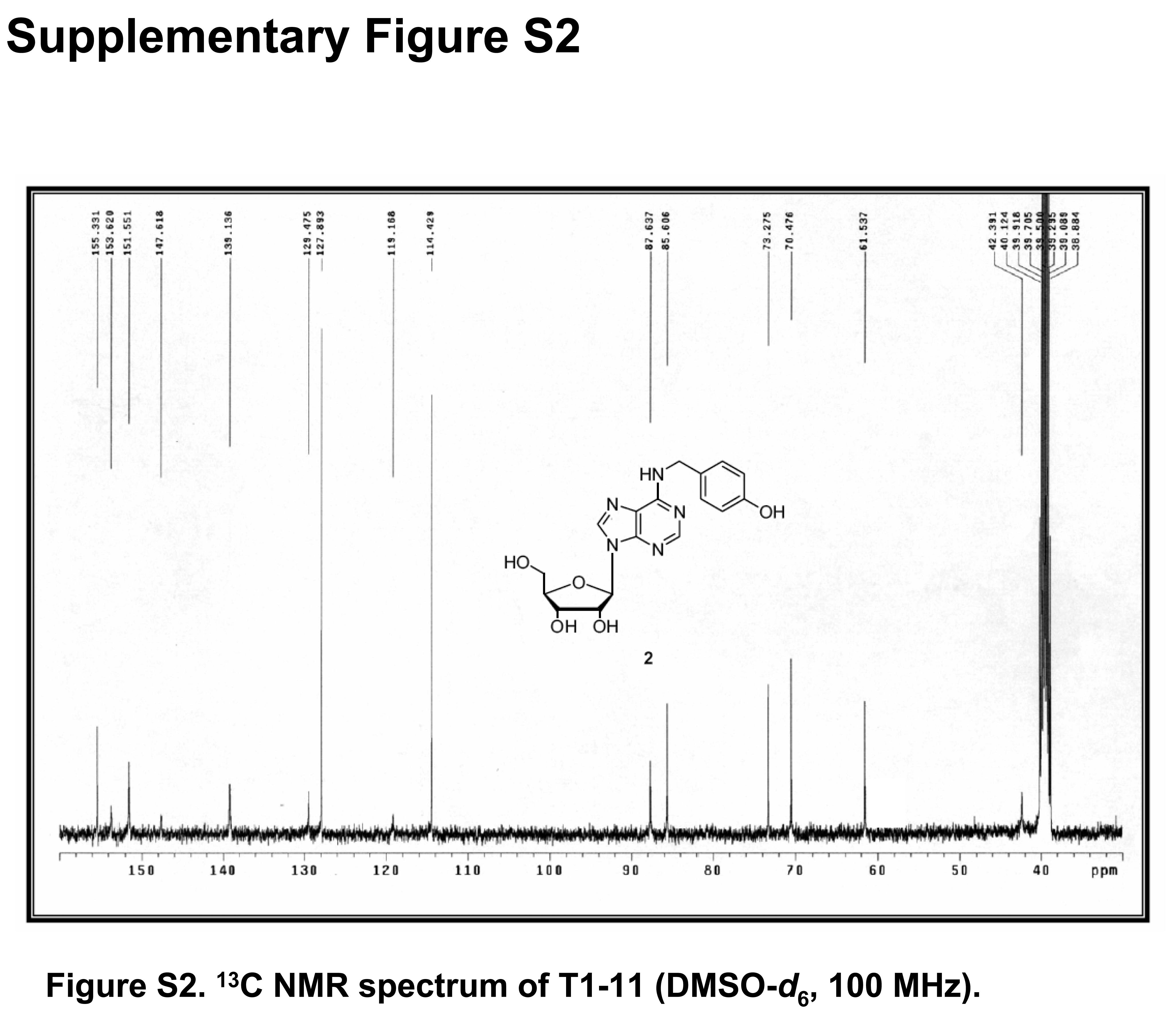

Supplement: Figure S2 — 13C NMR spectrum of T1-11 (DMSO- d 6, 100 MHz). (TIF) [file pone.0020934.s002.tif]

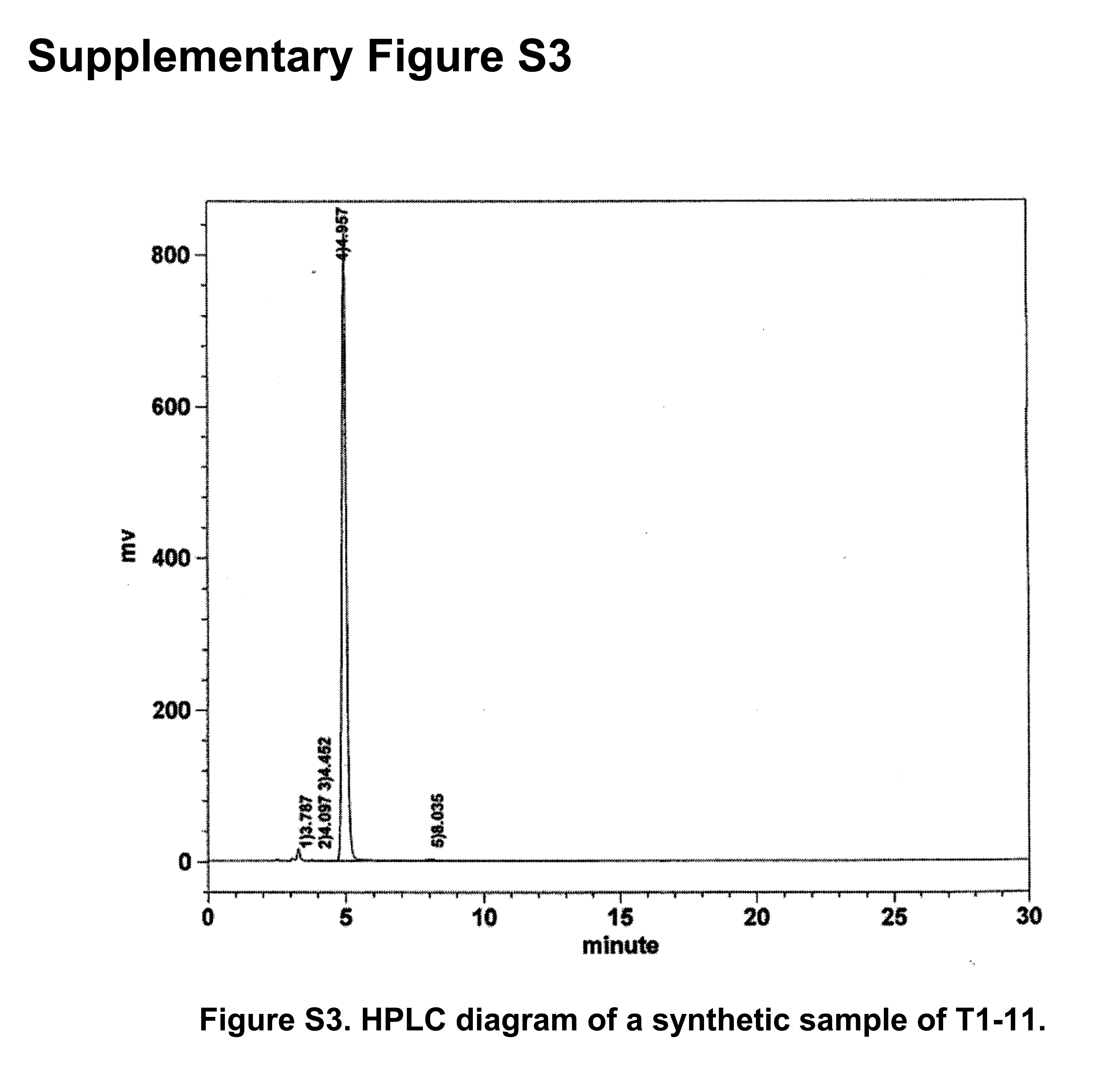

Supplement: Figure S3 — HPLC diagram of a synthetic sample of T1-11. (TIF) [file pone.0020934.s003.tif]

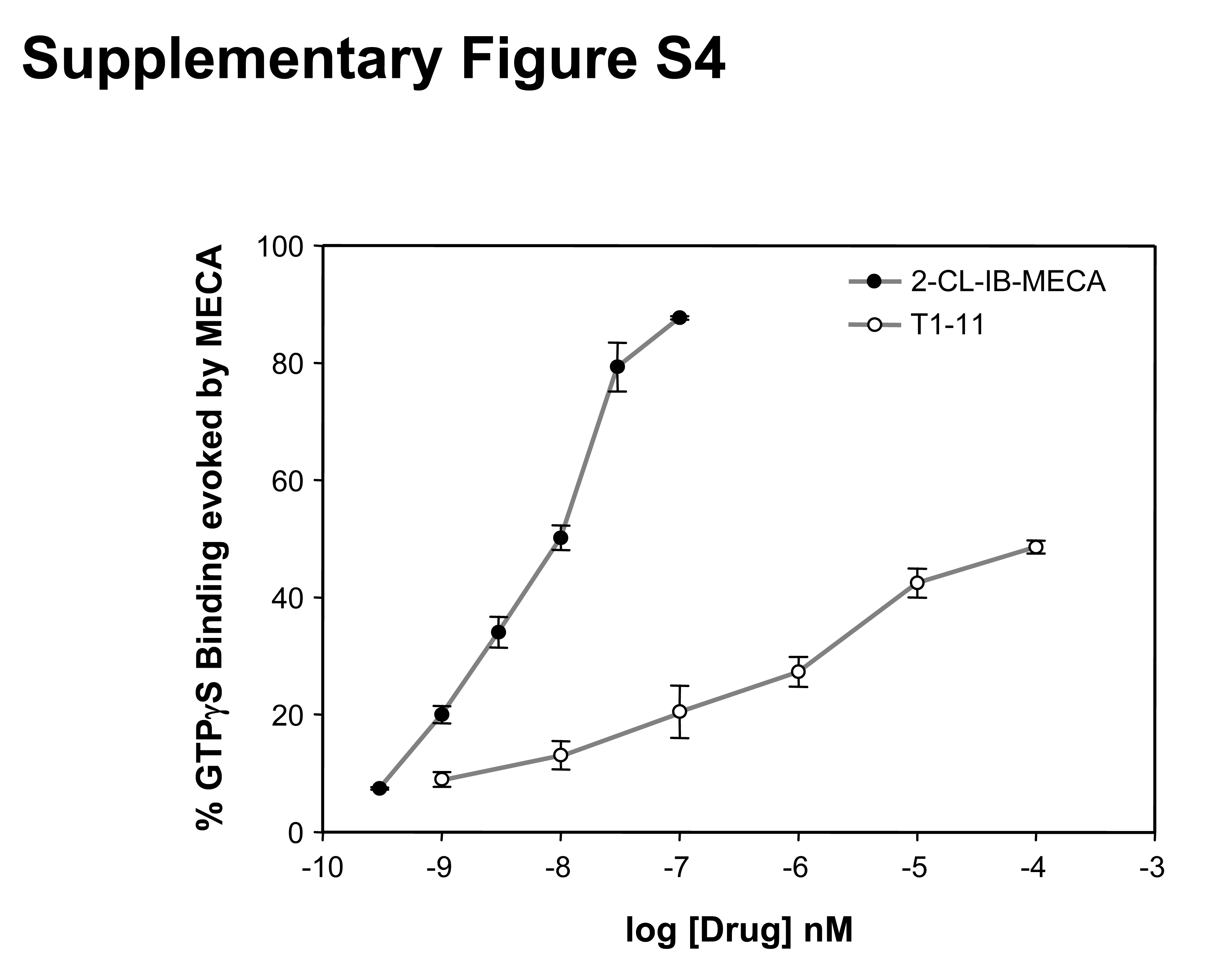

Supplement: Figure S4 — T1-11 binds to the A3 adenosine receptor (A3R) without evoking a significant binding of GTP. Membrane fractions collected from CHO-K1 cells expressing the human A3R were incubated with T1-11 at the indicated concentration and 35S-GTPγS (0.1 nM) for 30 min at 30°C. Relative GTPγS binding was defined as the percentage of 35S-GTPγS binding when compared with a selective agonist of the A3R (2-Cl-IB-MECA, 3 µM). (TIF) [file pone.0020934.s004.tif]

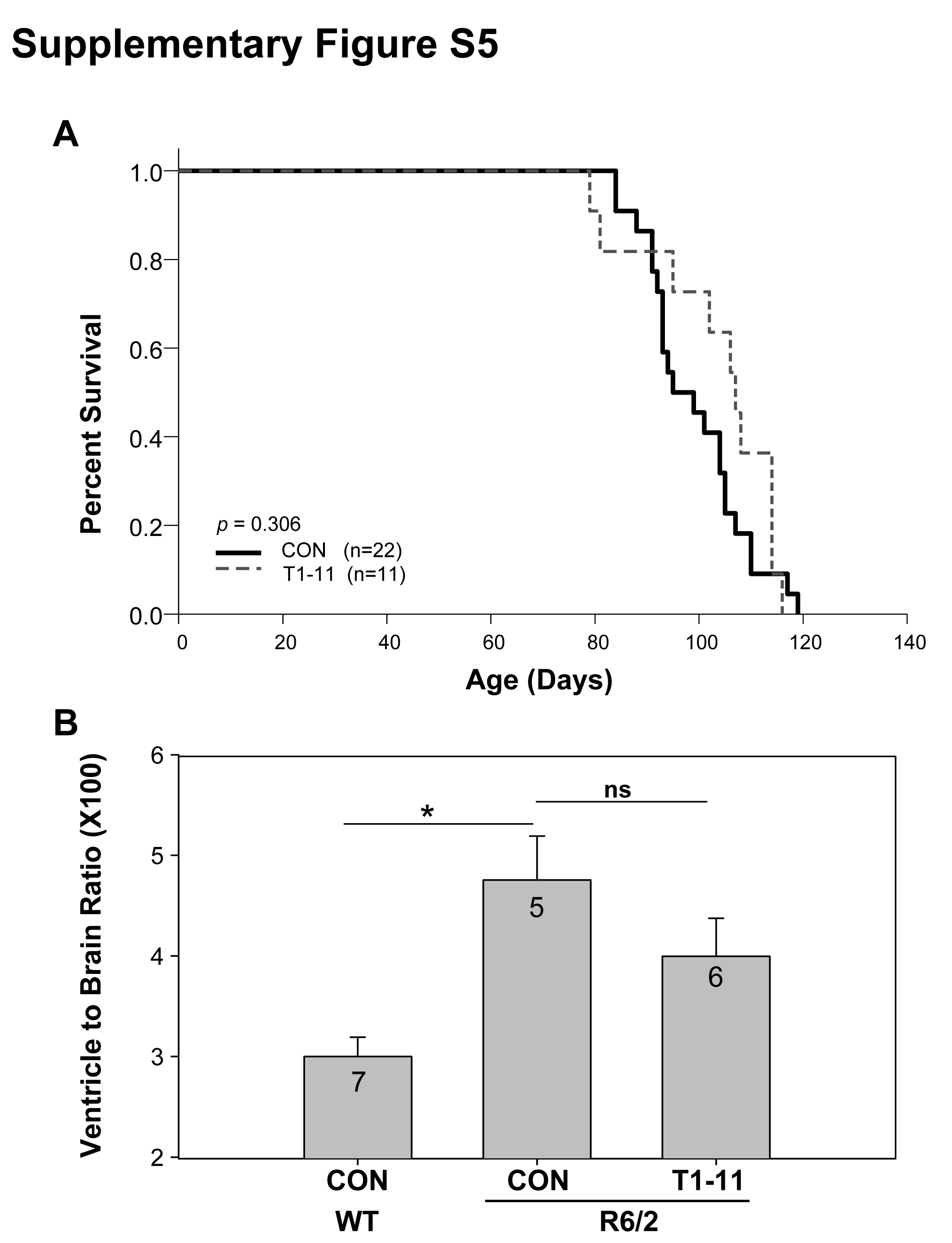

Supplement: Figure S5 — Effect of T1-11 on the shorten lifespan and enlarged ventricle of R6/2 mice. Animals were given the vehicle (1% DMSO; CON) or T1-11 (0.05 mg/ml)-containing drinking water from the age of 7 weeks. (A) Survival was assessed. Specific comparison to R6/2 mice treated with the vehicle (p = 0.306; Mantel-Cox test). (B) Five weeks after T1-11 treatment, 3D-lMRI was performed to determine the ventricle-to-brain ratio of the indicated animals as described. * p<0.05. (TIF) [file pone.0020934.s005.tif]

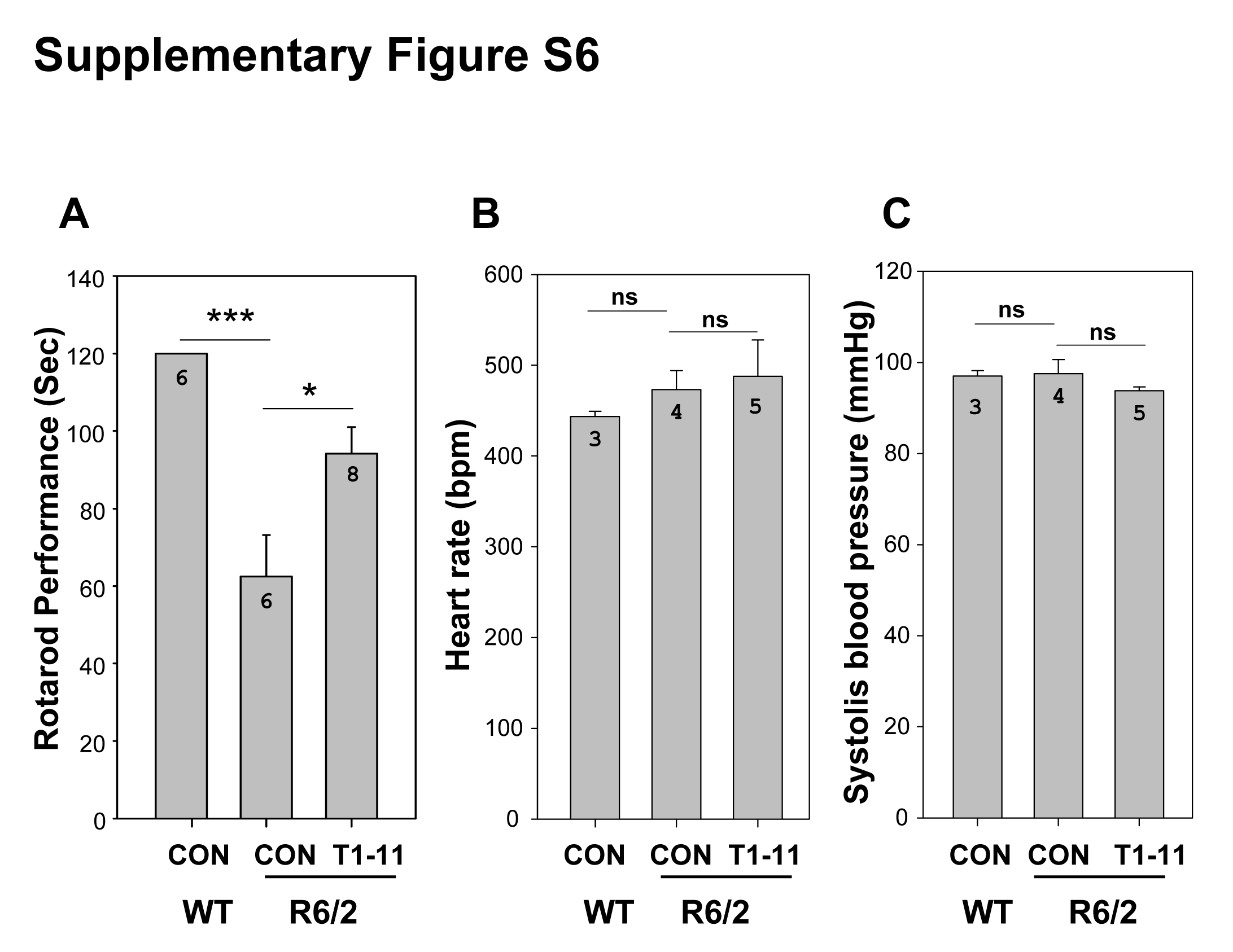

Supplement: Figure S6 — Treatment of R6/2 mice with T1-11 did not affect their heart rate and blood pressure. (A) T1-11 (125 µg/mouse/day) or vehicle (CON) was administrated subcutaneously to the indicated mice of 7 weeks old using ALZET osmotic minipumps for 6 weeks. Rotarod performance was assessed. (B, C) T1-11 (125 µg/mouse/day) was administrated subcutaneously to the indicated mice of 9 weeks old using ALZET osmotic minipumps for 48 h. Heart rate (B) and blood pressure (C) were determined by a tail-cuff method. * p<0.05. *** p<0.005. (TIF) [file pone.0020934.s006.tif]
