# Supplementary material for: A New Drug Design Targeting the Adenosinergic System for Huntington's Disease
Source: PLoS One. 2011 Jun 21;6(6):e20934. doi: 10.1371/journal.pone.0020934 (PMC3119665; doi:10.1371/journal.pone.0020934)
Supplement: Table S1 — Binding properties of T1-11 toward 208 proteins. (PDF) [file pone.0020934.s007.pdf]

Table S1 Binding properties of T1-11 toward 208 proteins.

Target	Species	n value	Concentration (µM)	% Inhibition
Adenosine A_1	human	2	10	23
**Adenosine A_{2A}**	**human**	**2**	**10**	**58**
Adenosine A2B	human	2	10	0
**Adenosine A_3**	**human**	**2**	**10**	**103**
Adrenergic α_1, Non-Selective	rat	2	10	-3
Adrenergic α_{1A}	rat	2	10	-14
Adrenergic α_{1B}	rat	2	10	3
Adrenergic α_{1D}	human	2	10	8
Adrenergic α_2, Non-Selective	rat	2	10	2
Adrenergic α_{2A}	human	2	10	-8
Adrenergic α_{2B}	human	2	10	2
Adrenergic α_{2C}	human	2	10	10
Adrenergic β, Non-Selective	rat	2	10	10
Adrenergic β_1	human	2	10	7
Adrenergic β_3	human	2	10	-1
Adrenomedullin AM1	human	2	10	11
Adrenomedullin AM2	human	2	10	9
Aldosterone	rat	2	10	2
Anaphylatoxin C5a	human	2	10	2
Androgen (Testosterone)	human	2	10	3
Angiotensin AT1	human	2	10	-5
Angiotensin AT2	human	2	10	3
APJ	human	2	10	21
Atrial Natriuretic Factor (ANF)	guinea pig	2	10	5
Benzodiazepine, Peripheral	rat	2	10	3
Bombesin BB1	human	2	10	-5
Bombesin BB2	human	2	10	3
Bombesin BB3	human	2	10	12
Bombesin, Non-Selective	rat	2	10	6
Bradykinin B_1	human	2	10	-8
Bradykinin B_2	human	2	10	-9
Calcitonin	human	2	10	15
Calcitonin Gene-Related, Peptide $CGRP_1$	human	2	10	6
Calcium Channel L-Type, Benzothiazepine	rat	2	10	-8
Calcium Channel L-Type, Dihydropyridine	rat	2	10	-4
Calcium Channel L-Type, Phenylalkylamin.	rat	2	10	-7
Calcium Channel N-Type	rat	2	10	1
Cannabinoid CB_1	human	2	10	-7
Cannabinoid CB_2	human	2	10	-2
Chemokine CCR1	human	2	10	-3
Chemokine CCR2B	human	2	10	0
Chemokine CCR4	human	2	10	5
Chemokine CCR5	human	2	10	-8
Chemokine CX3CR1	human	2	10	-1
Chemokine CXCR1/2 (IL-8, Non-Selective)	human	2	10	10
Chemokine CXCR2 (IL-$8R_B$)	human	2	10	0
Cholecystokinin CCK. (CCK_8)	human	2	10	-5
Cholecystokinin CCK. (CCK_A)	human	2	10	-3
Colchicine	rat	2	10	-2
Corticotropin Releasing Factor, CRF_1	human	2	10	1
Cyclooxygenase COX-2	human	2	10	0

Dopamine D_1	human	2	10	-13
Dopamine D_{2L}	human	2	10	13
Dopamine D_{2S}	human	2	10	10
Dopamine D_3	human	2	10	-7
Dopamine $D_{4.2}$	human	2	10	3
Dopamine $D_{4.4}$	human	2	10	8
Dopamine $D_{4.7}$	human	2	10	3
Dopamine D_5	human	2	10	2
Endothelin ETA	human	2	10	3
Endothelin ETB	human	2	10	2
Epidermal Growth Factor (EGF)	human	2	10	-5
Erythropoietin EPOR	human	2	10	3
Estrogen ER_α	human	2	10	32
Estrogen ER_β	human	2	10	40
G Protein-Coupled Receptor, GPR103	human	2	10	2
G Protein-Coupled Receptor, GPR8	human	2	10	15
$GABA_A$, Agonist Site	rat	2	10	4
$GABA_A$, Benzodiazepine, Central, Flunitrazepam	rat	2	10	6
$GABA_A$, Benzodiazepine, Central, Ro-15-1788	rat	2	10	1
$GABA_A$, Chloride Channel, TBOB	rat	2	10	-3
$GABA_A$, Chloride Channel, TBPS	rat	2	10	6
$GABA_A$, Non-Selective	rat	2	10	5
$GABA_{B1A}$	human	2	10	-1
$GABA_{B1B}$	human	2	10	-5
Gabapentin	rat	2	10	8
Galanin GAL1	human	2	10	0
Galanin GAL2	human	2	10	0
Glucocorticoid	human	2	10	-10
Glutamate, AMPA	rat	2	10	-9
Glutamate, Kainate	rat	2	10	11
Glutamate, NMDA, Agonism	rat	2	10	10
Glutamate, NMDA, Glycine	rat	2	10	7
Glutamate, NMDA, Phencyclidine	rat	2	10	-2
Glutamate, NMDA, Polyamine	rat	2	10	10
Glutamate, Non-Selective	rat	2	10	-1
Glycine, Strychnine-Sensitive	rat	2	10	10
Growth Hormone, Secretagogue (GHS, Ghrelin)	human	2	10	-4
Histamine H_1	human	2	10	1
Histamine H_2	human	2	10	-10
Histamine H_3	human	2	10	4
Histamine H_4	human	2	10	8
Imidazoline I_2, Central	rat	2	10	3
Imidazoline I_2, Peripheral	rat	2	10	10
Inositol Trisphosphate IP_3	rat	2	10	3
Insulin	rat	2	10	4
Interleukin IL-1	mouse	2	10	4
Interleukin IL-2	mouse	2	10	0
Interleukin IL-6	human	2	10	-3
Leptin	mouse	2	10	6
Leukotriene, BLT (LTB_4)	human	2	10	-6
Leukotriene, Cysteinyl $CysLT_1$	human	2	10	-4
Leukotriene, Cysteinyl $CysLT_2$	human	2	10	10

Lipoxygenase 12-LO	human	2	10	9
Lipoxygenase 15-LO	rabbit	2	10	14
Lipoxygenase 5-LO	human	2	10	25
Melanocortin MC1	human	2	10	3
Melanocortin MC3	human	2	10	3
Melanocortin MC4	human	2	10	5
Melanocortin MC5	human	2	10	1
Melatonin MT, Non-Selective	chicken	2	10	-3
Melatonin MT_1	human	2	10	-4
Melatonin MT_2	human	2	10	-20
Motilin	human	2	10	-2
Muscarinic M_1	human	2	10	-1
Muscarinic M_2	human	2	10	4
Muscarinic M_3	human	2	10	-6
Muscarinic M_4	human	2	10	9
Muscarinic M_5	human	2	10	0
Muscarinic, Non-Selective, Central	rat	2	10	12
Muscarinic, Oxotremorine-M	rat	2	10	-7
Neuromedin U NMU_1	human	2	10	-1
Neuromedin U NMU_2	human	2	10	5
Neuropeptide Y Y_1	human	2	10	6
Neuropeptide Y Y_2	human	2	10	4
Neurotensin NT_1	human	2	10	11
Neurotensin, Non-Selective	mouse	2	10	15
N-Formyl Peptide Receptor, FPR1	human	2	10	5
N-Formyl Peptide Receptor-like, FPRL1	human	2	10	5
Nicotinic Acetylcholine	human	2	10	6
Nicotinic Acetylcholine α1, Bungarotoxin	human	2	10	3
Nicotinic Acetylcholine α7, Bungarotoxin	rat	2	10	-1
Opiate δ (OP1, DOP)	human	2	10	19
Opiate κ (OP2, KOP)	human	2	10	6
Opiate μ (OP3, MOP)	human	2	10	5
Opiate, Non-Selective	rat	2	10	-13
Orexin OX_1	human	2	10	-13
Orexin OX_2	human	2	10	-7
Orphanin ORL_1	human	2	10	10
Phorbol Ester	mouse	2	10	9
Phosphodiesterase PDE1	bovine	2	10	26
Phosphodiesterase PDE1	human	2	10	17
Phosphodiesterase PDE10A1	human	2	10	1
Phosphodiesterase PDE3	human	2	10	0
Phosphodiesterase PDE4	human	2	10	0
Phosphodiesterase PDE5	human	2	10	4
Phosphodiesterase PDE6	human	2	10	21
Platelet Activating Factor (PAF)	human	2	10	7
Platelet-Derived Growth Factor (PDGF)	mouse	2	10	-4
Potassium Channel [K_A]	rat	2	10	1
Potassium Channel [K_{ATP}]	hamster	2	10	6
Potassium Channel [SK_{CA}]	rat	2	10	1
Potassium Channel HERG	human	2	10	-2
Progesterone	bovine	2	10	9
Progesterone PR-B	human	2	10	-8
Prostanoid CRTH2	human	2	10	0

Prostanoid DP	human	2	10	17
Prostanoid EP_2	human	2	10	10
Prostanoid EP_4	human	2	10	-6
Prostanoid, Thromboxane A_2 (TP)	human	2	10	1
Purinergic P_{2X}	rabbit	2	10	11
Purinergic P_{2Y}	rat	2	10	17
Rolipram	rat	2	10	-6
Ryanodine RyR3	rat	2	10	-1
Serotonin (5-Hydroxytryptamine) 5-HT_1, Non-Selective	rat	2	10	10
Serotonin (5-Hydroxytryptamine) 5-HT1A	human	2	10	9
Serotonin (5-Hydroxytryptamine) 5-HT1B	rat	2	10	3
Serotonin (5-Hydroxytryptamine) 5-HT_2, Non-Selective	rat	2	10	15
Serotonin (5-Hydroxytryptamine) 5-HT_{2A}	human	2	10	19
Serotonin (5-Hydroxytryptamine) 5-HT_{2B}	human	2	10	5
Serotonin (5-Hydroxytryptamine) 5-HT_{2C}	human	2	10	27
Serotonin (5-Hydroxytryptamine) 5-HT_3	human	2	10	5
Serotonin (5-Hydroxytryptamine) 5-HT_4	guinea pig	2	10	1
Serotonin (5-Hydroxytryptamine) 5-HT_{5A}	human	2	10	-7
Serotonin (5-Hydroxytryptamine) 5-HT_6	human	2	10	12
Serotonin (5-Hydroxytryptamine) 5-HT_7	human	2	10	2
Sigma σ_1	human	2	10	7
Sigma σ_2	rat	2	10	-3
Sigma, Non-Selective	guinea pig	2	10	-3
Sodium Channel, Site 2	rat	2	10	3
Somatostatin sst1	human	2	10	-3
Somatostatin sst2	human	2	10	9
Somatostatin sst3	human	2	10	15
Somatostatin sst4	human	2	10	5
Somatostatin sst5	human	2	10	4
Tachykinin NK_1	human	2	10	3
Tachykinin NK_2	human	2	10	3
Tachykinin NK_3	human	2	10	-3
Thyroid Hormone	rat	2	10	-9
Thyrotropin Releasing Hormone (TRH)	rat	2	10	-4
Transforming Growth Factor-β (TGF-β)	mouse	2	10	-1
**Transporter, Adenosine**	guinea pig	2	10	84
Transporter, Choline	rat	2	10	-10
Transporter, Dopamine	human	2	10	-6
Transporter, GABA	rat	2	10	8
Transporter, Glycin.e	rat	2	10	1
Transporter, Monoamine	rabbit	2	10	-6
Transporter, Norepinephrine (NET)	human	2	10	6
Transporter, Serotonin (5-Hydroxytryptamine) (SERT)	human	2	10	5
Tumor Necrosis Factor (TNF), Non-Selective	human	2	10	-2
Urotensin II	human	2	10	6
Vanilloid	rat	2	10	8
Vascular Endothelial Growth Factor (VEGF)	human	2	10	16
Vasoactive Intestinal Peptide VIP_1	human	2	10	2
Vasopressin V_{1A}	human	2	10	0
Vasopressin V_{1B}	human	2	10	-18
Vasopressin V_2	human	2	10	-5
Vitamin D_3	human	2	10	6
